# Machine learning and experimental validation identified autophagy signature in hepatic fibrosis

**DOI:** 10.3389/fimmu.2024.1337105

**Published:** 2024-02-28

**Authors:** Yushen Huang, Wen Luo, Zhijie Yang, Tian Lan, Xiaomou Wei, Hongwen Wu

**Affiliations:** ^1^ Department of Pharmacy, Liuzhou Workers Hospital, Liuzhou, Guangxi, China; ^2^ Department of Gastrointestinal Surgery, Liuzhou Workers Hospital, Liuzhou, Guangxi, China; ^3^ Department of Scientific Research, Liuzhou Workers Hospital, Liuzhou, Guangxi, China

**Keywords:** hepatic fibrosis, autophagy, immune, RB1CC1, ATG5, PARK2

## Abstract

**Background:**

The molecular mechanisms of hepatic fibrosis (HF), closely related to autophagy, remain unclear. This study aimed to investigate autophagy characteristics in HF.

**Methods:**

Gene expression profiles (GSE6764, GSE49541 and GSE84044) were downloaded, normalized, and merged. Autophagy-related differentially expressed genes (ARDEGs) were determined using the limma R package and the Wilcoxon rank sum test and then analyzed by GO, KEGG, GSEA and GSVA. The infiltration of immune cells, molecular subtypes and immune types of healthy control (HC) and HF were analyzed. Machine learning was carried out with two methods, by which, core genes were obtained. Models of liver fibrosis *in vivo* and *in vitro* were constructed to verify the expression of core genes and corresponding immune cells.

**Results:**

A total of 69 ARDEGs were identified. Series functional cluster analysis showed that ARDEGs were significantly enriched in autophagy and immunity. Activated CD4 T cells, CD56bright natural killer cells, CD56dim natural killer cells, eosinophils, macrophages, mast cells, neutrophils, and type 17 T helper (Th17) cells showed significant differences in infiltration between HC and HF groups. Among ARDEGs, three core genes were identified, that were ATG5, RB1CC1, and PARK2. Considerable changes in the infiltration of immune cells were observed at different expression levels of the three core genes, among which the expression of RB1CC1 was significantly associated with the infiltration of macrophage, Th17 cell, natural killer cell and CD56dim natural killer cell. In the mouse liver fibrosis experiment, ATG5, RB1CC1, and PARK2 were at higher levels in HF group than those in HC group. Compared with HC group, HF group showed low positive area in F4/80, IL-17 and CD56, indicating decreased expression of macrophage, Th17 cell, natural killer cell and CD56dim natural killer cell. Meanwhile, knocking down RB1CC1 was found to inhibit the activation of hepatic stellate cells and alleviate liver fibrosis.

**Conclusion:**

ATG5, RB1CC1, and PARK2 are promising autophagy-related therapeutic biomarkers for HF. This is the first study to identify RB1CC1 in HF, which may promote the progression of liver fibrosis by regulating macrophage, Th17 cell, natural killer cell and CD56dim natural killer cell.

## 1 Introduction

Hepatic fibrosis (HF) is a repair reaction of damage to liver tissue characterized by the deposition of extracellular matrix, which usually results from chronic liver injury related to alcoholism, hepatitis B virus infection, and fatty liver, among other causes (1). This disease can reduce the overall function of the liver—block hepatic blood flow, inhibit liver regeneration, and affect the liver’s ability to detoxify, store energy, and clear infections (2). If not properly managed, HF can progress to cirrhosis, eventually increasing the chances of developing liver cancer (3). Regardless of the main cause, liver fibrosis causes a higher mortality rate (4). Due to the complexity and heterogeneity of HF, the diagnosis and prognosis of this disease are poor. Therefore, it is imperative to further explore the pathogenesis and treatment of liver fibrosis.

Autophagy is a regulated mechanism that removes damaged or obsolete cellular cargoes and provides reuse of basic biomolecules (5). As a means of cytoplasmic degradation, autophagy is essential in maintaining hepatic metabolic homeostasis, indicating that targeting autophagy may be a potential approach in liver diseases (6). Autophagy can promote the activation of liver stellate cells by degrading lipid droplets, which is the key way of promoting the process of liver fibrosis (7). It follows that autophagy is closely related to liver fibrosis. Cao et al. reported that a specific PGE2/EP4 antagonist E7046 reduces M2 macrophage-mediated autophagy of liver stellate cells, subsequently alleviating liver fibrosis in nonalcoholic fatty liver disease mice (8). Research has shown that Fas/FasL is beneficial in relieving liver fibrosis via accelerating autophagy, while autophagy inhibitor impairs Fas/FasL-modulated hepatocyte apoptosis (9). Li et al. found that resolvin D1 relieves HF through decreasing autophagy-mediated hepatic stellate cell activation via AKT/mTOR signaling (10). However, a comprehensive bioinformatics study of autophagy characteristics in HF has not been reported.

The liver is an important immune organ that regulates its own repair by modulating immune cell activation (11). Studies indicate that an imbalance in the immune microenvironment of the liver is the main driver of liver fibrosis (12). Autophagy, which is regulated by a diverse array of immune cells, plays a role in managing the liver’s immune microenvironment (13, 14). Analysis of immune cell infiltration provides insights into the distribution and quantity of immune cells within liver tissue. Evaluating molecular subtypes allows for a deeper understanding of the functionality and characteristics of hepatic immune cells. Moreover, individual variations may exist in the immune systems of different patients. Gaining knowledge about these immunity types can aid in devising suitable treatments. Hence, a combined analysis of hepatic immunity can offer a more comprehensive understanding of the mechanisms underlying liver fibrosis.

In the present study, autophagy-related differentially expressed genes (ARDEGs) were determined using the limma R package and the Wilcoxon rank sum test. Functional enrichment analysis of ARDEGs was clarified by immune cell infiltration, molecular subtypes, and immune types. The risk model was then established by RF and SVM methods to screen out core genes. To verify the results of bioinformatics, we conducted a comprehensive experimental analysis. *In vivo*, a mouse liver fibrosis model was built to verify the expression of core genes and immune infiltration. Meanwhile, a cell model of HF was also set up to detect the expression and function of core genes.

## 2 Materials and methods

### 2.1 Data collection

We used the R package GEOquery (15) to acquire gene expression profile data for liver tissues with HF from the GEO database (16) including GSE6764 (17), GSE49541 (18, 19) and GSE84044 (20) (**SupplementaryTable S1**). The GSE6764 dataset contained 10 HC and 65 HF samples, the GSE49541 dataset contained 72 HF samples, and the GSE84044 dataset contained 124 HF samples. All datasets were based on the GPL570 [HG-U133_Plus_2] Affymetrix Human Genome U133 Plus 2.0 Array platform. These datasets were merged, followed by de-batching, normalization, and probe annotation. The limma R package (21) was used to obtain gene expression from the expression matrix containing 10 HC samples and 261 HF samples.

### 2.2 Determination of ARDEGs in HF

To assess the effect of autophagy in HF, we downloaded 232 autophagy related genes (ARGs) from the Human Autophagy Database (HADb, http://www.autophagy.lu/) (22–24). The DEGs between the HC and HF groups were screened and visualized as volcano plots. The upregulated genes were determined by |logFC| > 1 and P < 0.01, and the downregulated genes were determined by |logFC| <1 and P < 0.01. The Wilcoxon rank-sum test was used to acquire the 69 most significant ARDEGs, which were then visualized using the pheatmap R package.

### 2.3 Immune infiltration analysis between HC and HF

Single-sample gene set enrichment analysis (ssGSEA) (25) was used to estimate immune cell infiltration between the HC and HF groups using data obtained from Charoentong (26). Based on the results of ssGSEA, we calculated Spearman’s correlation coefficient between immune cell infiltration and ARDEGs expression, which was displayed using a correlation heat map. A FRIENDS analysis was conducted to investigate the importance of ARDEGs using the GOSemSim R package (27, 28).

### 2.4 Gene ontology and Kyoto encyclopedia of genes and genomes analysis

We used the ClusterProfiler package (29) to conduct Gene Ontology (GO) functional enrichment and Kyoto Encyclopedia of Genes and Genomes (KEGG) biological pathway analyses for the ARDEGs, with P < 0.05 considered statistically significant.

### 2.5 Disease ontology analysis

To explore the enrichment of the ARDEGs in HF, we used the DOSE package (30) for Disease Ontology (DO) analysis, which estimates correlations between gene expression and disease state to elucidate molecular mechanisms. Statistical significance was set at P < 0.05.

### 2.6 Gene set variation analysis and GSEA

Using the gene set variation analysis (GSVA) package (31), we conducted a GSVA (a nonparametric unsupervised method) to evaluate ARDEGs enrichment in the “c2.cp.kegg.v7.5.1.symbols.gmt” gene set obtained by MSigDB (v7.5.1) (32); the threshold was set to |logFC| > 0.1 and P < 0.05.

Using the ClusterProfiler R package, we conducted a GSEA (33) to assess the distribution of ARDEGs in a phenotypic correlation sequencing gene table to determine their contributions to the HF phenotype. The “c2.cp.v7.5.1.symbols.gmt” gene set was acquired from the MSigDB database (v7.5.1) (32). Statistical significance was set at P < 0.05.

### 2.7 Construction of molecular subtypes

The ConsensusClusterPlus R package (34) was applied to divide samples of the HF group into two molecular subtypes based on unsupervised clustering of ARDEG expression. The ggplot2 R package was used to perform a Principal Component Analysis (PCA). Differences in ARDEG expression among the different subtypes were explored using boxplots.

### 2.8 Immunological features among different molecular subtypes

ssGSEA (25) was used to calculate and quantify immune cell infiltration between the two molecular subtypes using data obtained from Charoentong (26). A boxplot was constructed using these results to explore the infiltration status of immune cells in the different molecular subtypes.

### 2.9 Construction of immune typing

Based on the results of immune cell infiltration between HF samples, non-negative matrix factorization (NMF) was used to classify the HF samples into different immunophenotypes. A volcano map was used to visualize DEGs expression between different immune subtypes, and a heat map was used to evaluate the variation in immune cell infiltration between DEGs in the different immune subtypes.

### 2.10 Construction of risk model

We assessed the performance of RF and SVM methods in constructing the HF risk model. The residual, receiver operating characteristic (ROC) curve, and area under the curve (AUC) were used to compare model performance. The method with the smallest residual and largest AUC was selected for model construction. To screen ARDEGs with significant influence on HF occurrence, we calculated Gini indices to measure the effect of each variable on the heterogeneity of observed values at each node of the classification tree. The lrm model algorithm was used to screen candidate genes for further model construction. A nomogram was used to visualize the impact of each ARDEG on HF risk and identify the parameters of the final risk model. Calibration curves and a decision curve analysis (DCA) were used to evaluate the performance of the final HF risk model.

### 2.11 Establishment of PPI network

The STRING database (35) was used to construct the PPI network of the ARDEGs. ARDEG data were imported into the STRING database, the confidence value was set to 0.4, and the results were visualized using Cytoscape (v3.9.1). We applied the CytoHubba plugin (36) to analyze elements in the PPI network, and hub genes were screened according to the MCC score.

### 2.12 Network analysis of core genes with micro RNAs, transcription factors, and RNA-binding proteins

The MiRWalk database (37) (http://mirwalk.umm.uni-heidelberg.de/) was used to obtain micro RNAs (miRNAs) associated with the core genes. Transcription factors (TFs) control gene expression by interacting with target genes during the post-transcription phase. To analyze their association, the TRRUST database (38) (https://www.grnpedia.org/trrust/) was used to identify TFs that bind to the core genes. RNA-binding proteins (RBPs) are a class of molecules that recognize specific RNA-binding domains. We used the starBase database (https://starbase.sysu.edu.cn/) to identify RBPs associated with the core genes. The networks of core genes with miRNAs, TFs, and RBPs were visualized using Cytoscape (v3.9.1).

### 2.13 Construction of mouse liver fibrosis model

Both male and female C57BL/6 mice (SPF grade, 6-8 weeks of age, weighing 20 ± 2g) were obtained from the Experimental Animal Center of Guangxi Medical University (Guangxi, China). After 1 week with standard chow and water provided ad libitum, the mice were randomly divided into normal and model groups (n = 15 per group). The mice in the model group were intraperitoneally injected with 0.5 mL/100 g 20% CCl4 oil solution three times a week for 10 weeks, and mice in the normal group were intraperitoneally injected with the same amount of oil solution. After the last administration, all animals were sacrificed under pentobarbitone sodium anesthesia and liver tissue samples were obtained for further analysis.

### 2.14 Sirius staining

Mouse liver tissues were fixed in 4% paraformaldehyde for 48 h and subsequently embedded in paraffin wax. The paraffin-embedded tissues were cut into 4-μm slices and stained with Sirius staining according to standard procedures.

### 2.15 Reverse transcription-polymerase chain reaction

Total RNA from liver tissues was isolated using the TRIzol method. cDNA—the PCR template—was produced by reverse transcription using a one-step RT Kit (Takara Biotechnology, Dalian, China). PCR was performed using an SYBR Green Quantitative PCR kit (Takara Biotechnology). ATG5, PARK2, and RB1CC1 mRNA levels were measured and normalized to the levels of β-actin. The RT-PCR conditions were as follows: a single step at 95°C for 30 s, followed by 40 cycles at 95°C for 5 s and 60°C for 30 s. Primer sequences for the genes used in the experiment are listed in **Supplementary Table S2**.

### 2.16 Immunohistochemistry

Immunohistochemical staining of liver tissue was performed as described in previous study (39). Following antibodies were used for immunohistochemistry: F4/80 (1:1000, Servicebio), IL-17 (1:1000, Servicebio) and CD56 (1:1000, Servicebio).

### 2.17 Cell culture and RNAi assays

LX2 cells were cultured in a constant temperature incubator at 37 °C and 5% CO2. When the cell density reached 80%, 2 ng/mL PDGF was added. After incubation for 2 h, RB1CC1 small interfering RNA (siRNA) was transfected into LX-2 cells using Lipofectamine 2000 (Invitrogen). RB1CC1 siRNA was designed and synthesized by GenePharma.

### 2.18 Colony formation analysis

LX2 cells were observed daily under a microscope, and the medium was changed if the metabolites in the cells increased. After 8–10 d, the medium was discarded and the cells stained with crystal violet.

### 2.19 Acridine orange/ethidium bromide staining

Pancreatic enzymes were added for digestion of LX2 cells, followed by centrifugation. Staining was performed using an acridine orange/ethidium bromide staining kit (Shanghai Yuanye Bio-Technology Co., Ltd.).

### 2.20 Tissue microarray analysis

The tissue microarray analysis was conducted by Xi’an Bioaitech Co. Ltd of China. The chip number was DP087Lv01. The RB1CC1 expression levels were detected by immunohistochemistry.

### 2.21 Statistical analysis

All calculations and statistical analyses were performed using R software (https://www.r-project.org/, v4.0.2). For the comparison of two sets of continuous variables, we used an independent Student’s t-test for normally distributed variables and a Mann-Whitney U test (Wilcoxon rank sum test) for non-parametric variables. All tests were two-tailed, and statistical significance was set at P < 0.05.

## 3 Results

### 3.1 ARDEGs between the HC and HF groups

**Figure 1:**
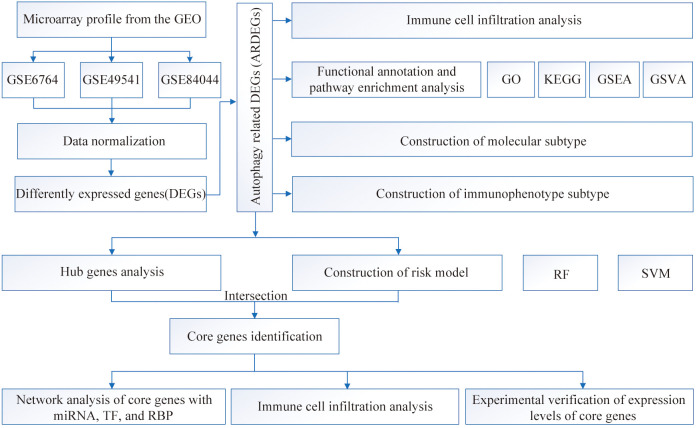
Flowchart of the overall analysis.

**Figure 2:**
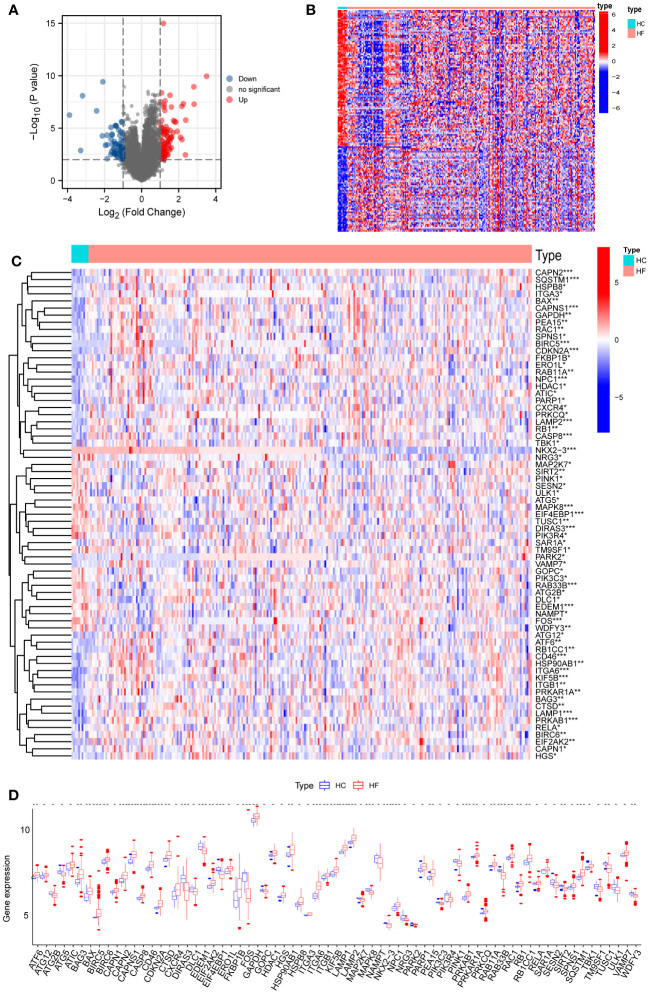
Differential expressions of ARDEGs between HF and HC groups. **(A)** Volcano plot of DEGs. The X-axis is log2FoldChange, and the Y-axis is -log10 (P value). **(B)** Heatmap of DEGs. Red dots represent up-regulated DEGs, and blue dots show down-regulated DEGs. **(C)** Heatmap of ARDEGs. Red dots represent up-regulated DEGs, and blue dots indicate down-regulated DEGs. **(D)** Boxplot of ARDEGs. * P < 0.05, ** P < 0.01, and *** P < 0.001.

**Figure 1** presents the workflow of our, mainly bioinformatic, analyses. The HC and HF groups were probe annotated and standardized (**Supplementary Figure S1**). Using the limma R package, we screened 160 DEGs, of which 91 were upregulated and 69 were downregulated (**Figures 2A, B**). The Wilcoxon rank sum test identified 69 ARDEGs (**Figures 2C, D**). Correlations among the ARDEGs were calculated and displayed using a correlation graph (**Supplementary Figure S2**).

### 3.2 Immune infiltration between HC and HF groups

**Figure 3:**
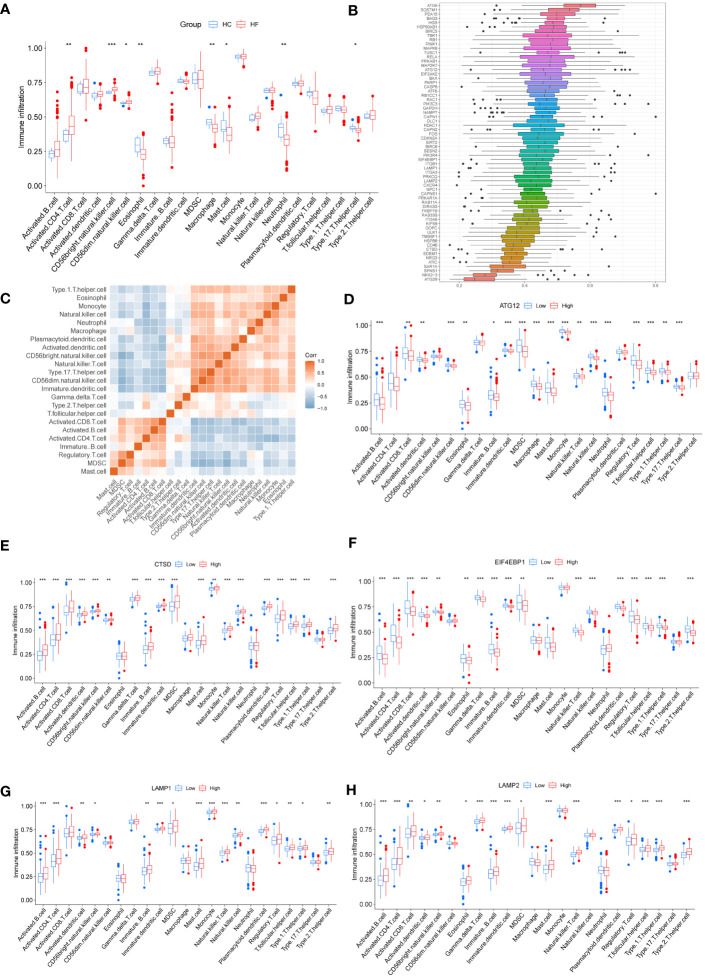
Immune infiltration analysis between HC and HF groups. **(A)** Differences in immune cell infiltration between HF and HC groups. Blue represents the HC group and red indicates the HF group. **(B)** Boxplot of FRIENDS analysis of ARDEGs. Each box represents an ARDEGS, the middle vertical line shows the median value of its expression, the two ends of the line indicate the maximum and minimum values, and the black dots represent outliers. **(C)** Correlation heatmap of immune cells in HF group. Red represents a positive correlation and blue represents a negative correlation. D-H Immune cell infiltration under different expression levels of some ARDEGs. Blue represents low expression group and red indicates high expression group. ATG12 **(D)**, CTSD **(E)**, EIF4EBP1 **(F)**, LAMP1 **(G)**, LAMP2 **(H)**. * P < 0.05, ** P < 0.01, and *** P < 0.001.

We used ssGSEA to analyze the levels of immune cell infiltration between HC and HF groups and found that activated CD4 T cells, CD56bright natural killer cells, CD56dim natural killer cells, eosinophils, macrophages, mast cells, neutrophils, and type 17 T helper (Th17) cells showed significant differences in infiltration between groups (**Figure 3A**). FRIENDS analysis revealed the importance of ARDEGs (**Figure 3B**). The correlation between immune cell infiltration in the HF group was visualized using a correlation heatmap (**Figure 3C**). **Figure 3D–H** shows the difference in the levels of infiltration of immune cells between different expression levels of some ARDEGs in HF.

### 3.3 GO and KEGG enrichment analyses

**Figure 4:**
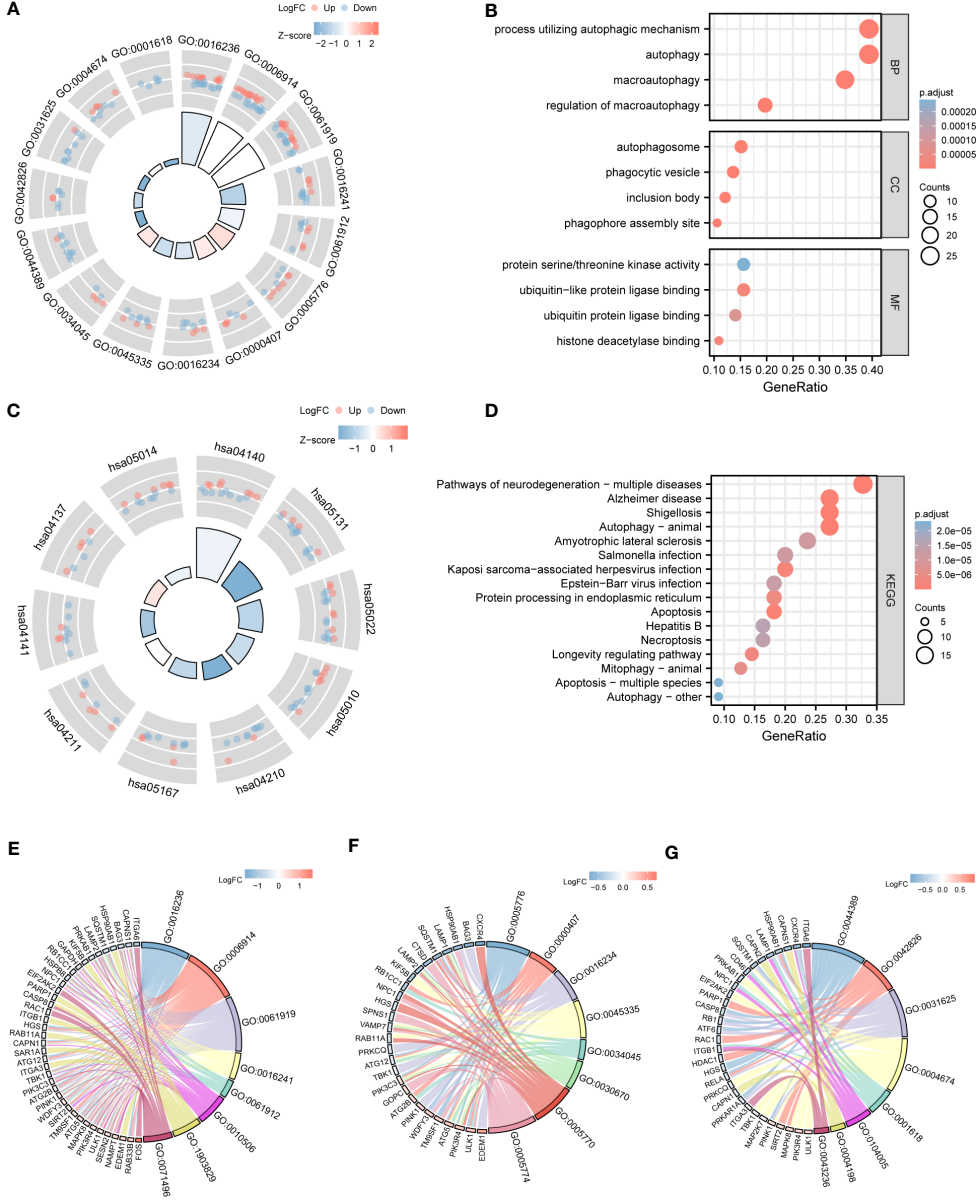
GO and KEGG enrichment analysis. **(A)** Circle plot of GO enrichment analysis of ARDEGs. The outer circle is GO terms; red dots show upregulated genes, and blue dots show downregulated genes. The quadrilateral color represents the zscore of the GO terms; red color indicates that the z-score is positive and the corresponding GO terms are more likely to be activated; while blue color indicates that the z-score is negative and the corresponding GO terms are more likely to be inhibited. **(B)** Bubble plot of GO enrichment analysis of ARDEGs. The X-axis is GeneRatio, and the Y-axis is Pathway name. The node size represents the number of genes enriched in the Go term, and the node color represents p.value. **(C)** Circle plot of KEGG enrichment analysis of ARDEGs. **(D)** Bubble plot of KEGG enrichment analysis of ARDEGs. **(E–G)** are chord diagram of BP, CC and MF functional enrichment analysis, respectively.

GO analysis revealed that ARDEGs were enriched in the biological processes (BP) of macroautophagy, autophagy, process utilizing autophagic mechanism, and regulation of macroautophagy (**Figures 4A, B, E**, **Supplementary Table S3**); the cellular components (CC) of autophagosome, phagophore assembly site, inclusion body, and phagocytic vesicle (**Figures 4A, B, F**, **Supplementary Table S3**); and the molecular functions (MF) of ubiquitin-like protein ligase binding, histone deacetylase binding, ubiquitin protein ligase binding, and protein serine/threonine kinase activity (**Figures 4A, B, G**, **Supplementary Table S3**). KEGG analysis showed that ARDEGs were enriched in pathways such as autophagy-animal, shigellosis, pathways of neurodegeneration-multiple diseases, and Alzheimer’s disease (**Figures 4C, D**, **Supplementary Table S4**). These results demonstrated that ARDEGs were mainly enriched in autophagy phenotypes.

### 3.4 GSEA, GSVA, and DO analysis

**Figure 5:**
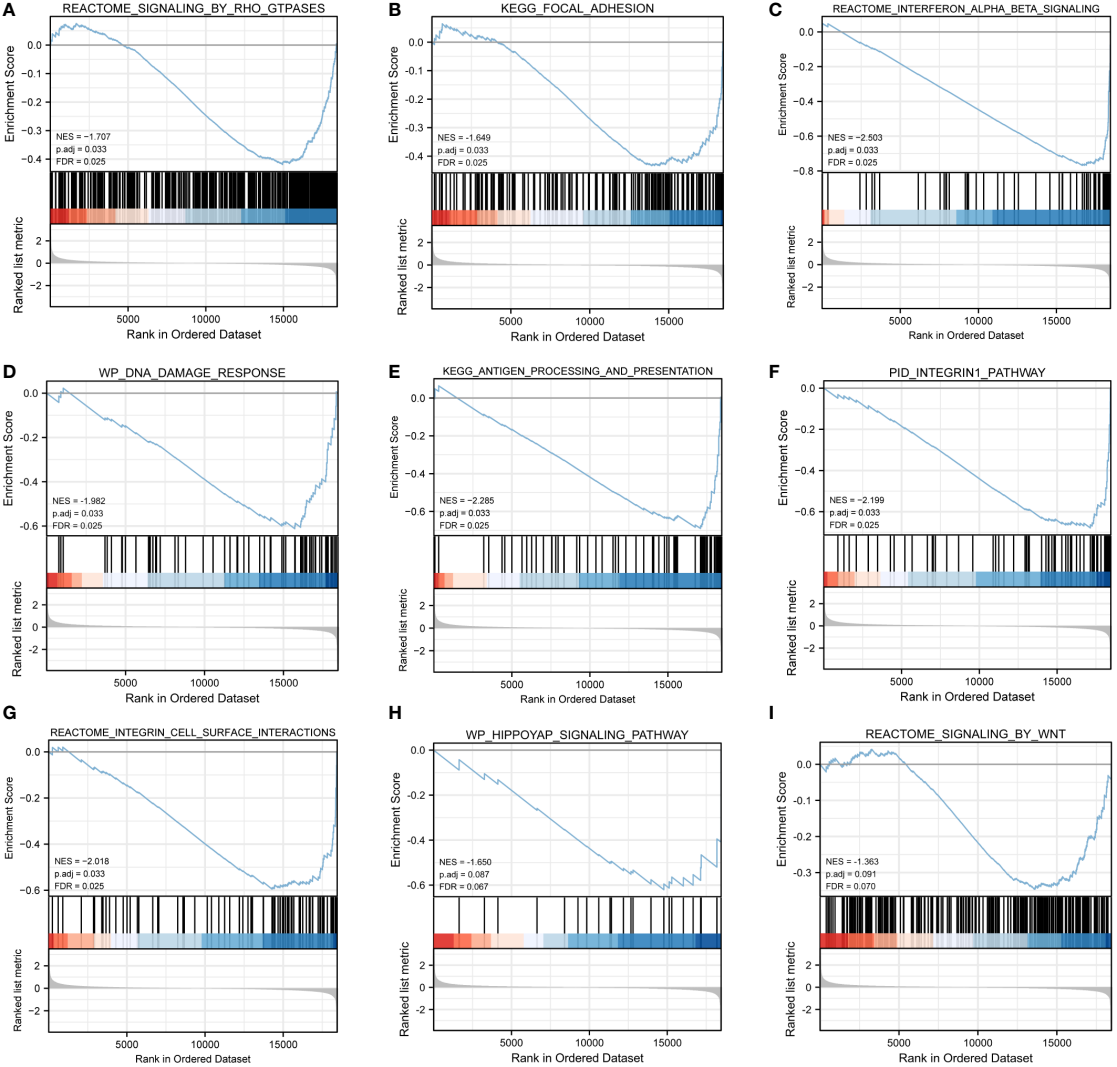
GSEA. **(A–I)** GSEA results showed that ARDEGs mainly annotated in REACTOME SIGNALING BY RHO GTPASES **(A)**, KEGG FOCAL ADHESION **(B)**, REACTOME INTERFERON ALPHA BETA SIGNALING **(C)**, WP DNA DAMAGE RESPONSE **(D)**, KEGG ANTIGEN PROCESSING AND PRESENTATION **(E)**, PID INTEGRIN1 PATHWAY **(F)**, REACTOME INTEGRIN CELL SURFACE INTERACTIONS **(G)**, WP HIPPOYAP SIGNALING PATHWAY **(H)**, REACTOME SIGNALING BY WNT **(I)**. False discovery rate (FDR) < 0.25 and P < 0.05 were considered as significant enrichment.

We also conducted GSEA, GSVA, and DO analyses to determine differences in physiological pathways and effects between the HF and HC groups. The GSEA showed that some physiological processes (**Figure 5**, **Supplementary Table S5**) differed dramatically, such as reactome signaling by Rho-GTPases (**Figure 5A**), KEGG focal adhesion (**Figure 5B**), reactome interferon alpha beta signaling (**Figure 5C**), WP DNA damage response (**Figure 5D**), KEGG antigen processing and presentation (**Figure 5E**), PID integrin 1 pathway (**Figure 5F**), reactome integrin cell surface interactions (**Figure 5G**), WP HIPPOYAP signaling pathway (**Figure 5H**), and reactome signaling by WNT (**Figure 5I**). These processes are all closely related to the hepatic immune system.

The results of GSVA suggested that ARDEGs were enriched in some important pathways, most of which were also liver immune microenvironment pathways, including KEGG endocytosis, KEGG lysosome, KEGG antigen processing and presentation, KEGG extracellular matrix receptor interaction, KEGG autoimmune thyroid disease, and KEGG P53 signaling pathway (**Supplementary Figure S3A**, **Supplementary Table S6**). DO analysis revealed that ARDEGs were annotated in several disease pathways, including hepatitis, pancreatic cancer, breast carcinoma, benign mesothelioma, and head and neck squamous cell carcinoma (**Supplementary Figure S3B**). These results suggest that these ARDEGs might have a strong association with autophagy and immunity.

### 3.5 Construction of HF-associated molecular subtypes

**Figure 6:**
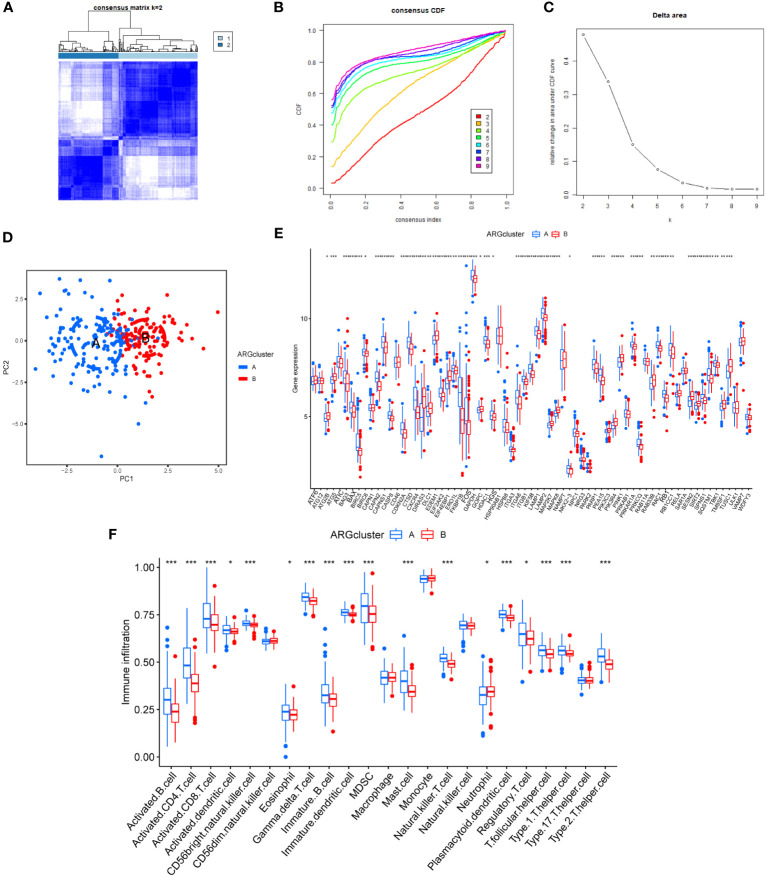
Construction of HF-associated molecular subtypes and their immunological features. **(A–C)**. Unsupervised cluster analysis based on the expression of ARDEGs in HF samples. **(D)** PCA plot of different molecular subtypes. **(E)** Boxplot of ARDEGs expression difference among different molecular subtypes. **(F)** Immune cell infiltration among different molecular subtypes. * P < 0.05, ** P < 0.01, and *** P < 0.001.

All HF samples were classified into two subtypes by unsupervised clustering: ARG cluster A and ARG cluster B, respectively (**Figures 6A–C**). The PCA and boxplot revealed that these clusters had distinct distributions (**Figures 6D, E**). The ssGSEA showed that activated B cells, activated CD4 T cells, activated CD8 T cells, CD56bright natural killer cells, and gamma delta T cells showed significant differences in infiltration between the subtypes (**Figure 6F**).

### 3.6 Determination of immunophenotyping

**Figure 7:**
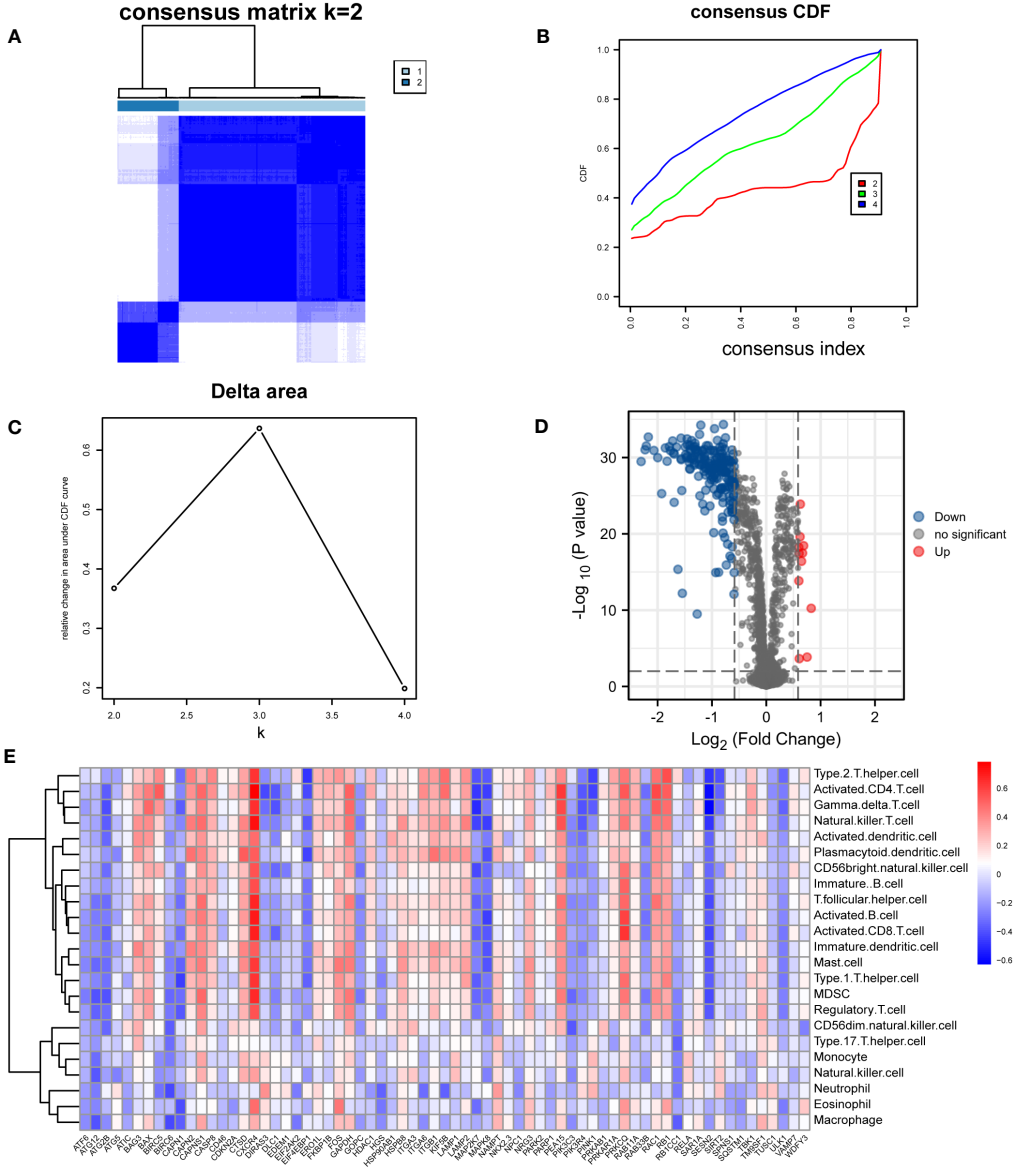
Construction of Immunophenotyping. **(A–C)** Construction of HF immunotyping based on immune cell infiltration. **(A)** Consensus clustering matrix for k = 2. **(B)** Consensus clustering cumulative distribution function (CDF) for k = 2–4. **(C)** Relative change in area under CDF curve for k = 2–4. **(D)** Volcano plot based on DEGs analysis among immunotyping. **(E)** Correlation heatmap between immune cells and DEGs of different immunotyping.

To further assess the differences in immune cell infiltration, the HF samples were divided into two immunophenotypes: cluster A and cluster B (**Figures 7A–C**). A total of 208 DEGs, 197 downregulated and 11 upregulated genes, were screened among the immunophenotypes (**Figure 7D**). A correlation heatmap was drawn to assess the involvement of DEGs in immune cell infiltration based on immunophenotype (**Figure 7E**). All ARDEGs were among the DEGs based on immunophenotype, suggesting a close relationship between ARDEGs and different immunophenotypes.

### 3.7 Identification of hub genes

**Figure 8:**
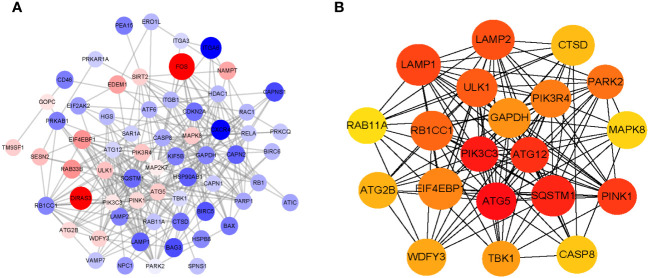
Identification of Hub genes. **(A)** Analysis of protein-protein interaction (PPI) of ARDEGs. The bigger the circle size, the higher expression multiple of ARDEGs. Red dots represent up-regulated ARDEGs, and blue dots show down-regulated ARDEGs. **(B)** The top 20 hub genes analysis using CytoHubba.

We used the STRING database and Cytoscape software to build the PPI network of ARDEGs (**Figure 8A**). The cytoHubba plugin identified the top 20 hub genes based on the MCC score, including ATG5, PIK3C3, SQSTM1, ATG12, PINK1, LAMP1, LAMP2, ULK1, RB1CC1, PARK2, PIK3R4, EIF4EBP1, GAPDH, TBK1, WDFY3, ATG2B, CTSD, CASP8, MAPK8, and RAB11A (**Figure 8B**).

### 3.8 Construction of the HF risk model

**Figure 9:**
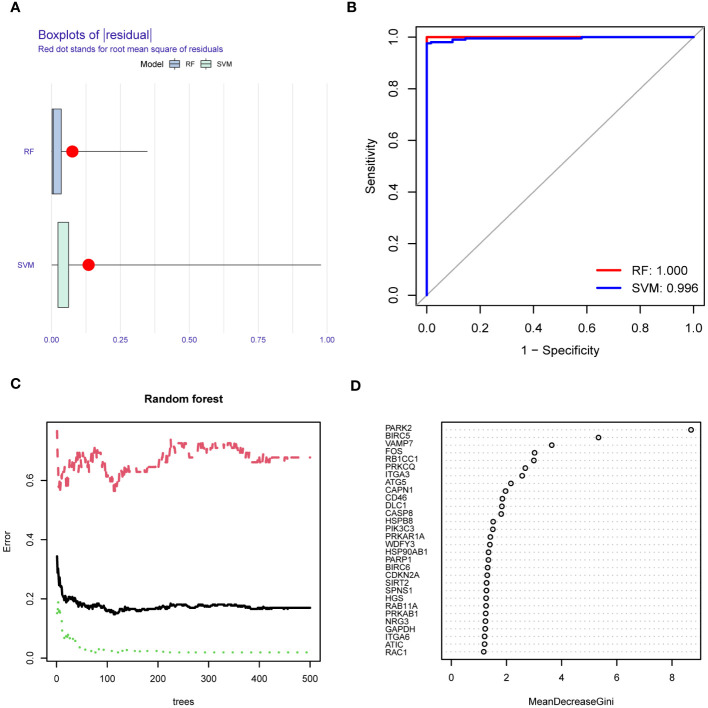
Comparison of the construction methods of the risk models. **(A)** Residual histogram of random forest (RF) and support vector machine (SVM). Blue represents RF and green indicates SVM. **(B)** Receiver Operating Characteristic Curve (ROC) of RF and SVM. Red represents RF and blue indicates SVM. Area Under Curve (AUC) of RF is 1.00 and that of SVM is 0.996. **(C)** RF diagram of risk model. Green represents training set, red shows test set, and black indicates all sample data sets. **(D)** GINI index of ARDEGs. The Y-axis is gene names and the X-axis is the decline degree of the GINI index of corresponding gene.

**Figure 10:**
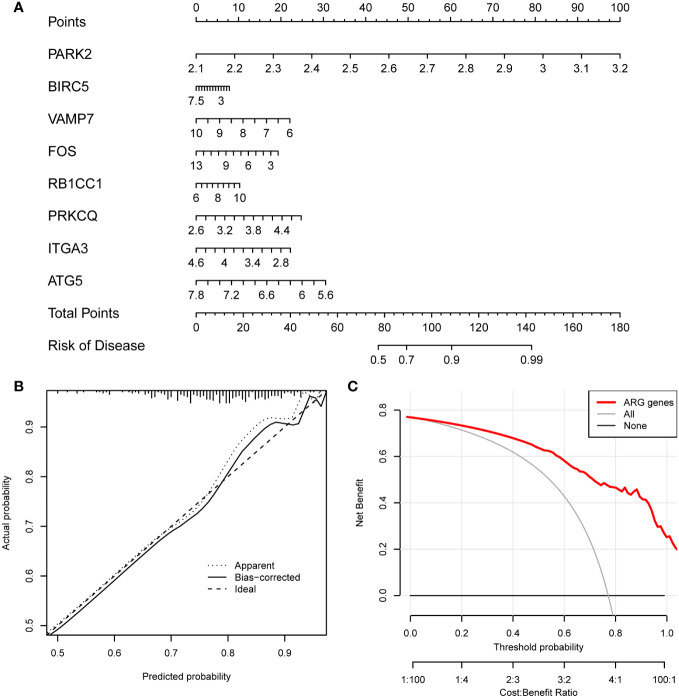
Construction of the risk model. **(A)** Nomogram of the risk model. The expression level of each gene corresponds to a score, and the final total score corresponds to the risk of HF. **(B)** Calibration curve of the risk model. Diagonal is ideal curve, and floating point line and solid line are the model curves before and after correction. The closer the floating point line and solid line fit to the diagonal, the better the model discrimination. **(C)** Decision curve of clinical utility. Red is the decision curve after the inclusion of screened ARDEGs, gray is the decision curve with all genes included, and black is the decision curve without any genes included. It can be seen that the model will bring higher clinical benefits after the inclusion of screened ARDEGs.

We determined the optimal model construction method between RF and SVM. The RF method had a smaller residual, suggesting a better consistency in data fit than the SVM method (**Figure 9A**). Given that the accuracy of the risk model based on the RF method was higher than that with the SVM method (**Figure 9B**), we selected the RF method to construct the HF risk model. A RF diagram was drawn with 30 ARDEGs (**Figure 9C**) and the GINI index was used to assess the contribution of each ARDEG in HF risk (**Figure 9D**). Using the lrm model algorithm, we screened eight ARDEGs, including PARK2, BIRC5, VAMP7, FOS, RB1CC1, PRKCQ, ITGA3, and ATG5. The nomogram was used to assess the impact of each ARDEG on HF risk and evaluate the HF risk of each sample based on the impact scores (**Figure 10A**). The calibration curve of the risk model exhibited good consistency (**Figure 10B**). The decision curve of clinical utility showed that the predictive power of the HF risk model based on the eight ARDEGs was significantly greater than that of the model based on all DEGs (**Figure 10C**), indicating that our risk model identified ARDEGs with the highest correlation with HF risk.

### 3.9 Network analysis of core genes with miRNAs, TFs, and RBPs

Of the 20 hub genes and eight risk model ARDEGs, we identified three intersecting genes, including ATG5, PARK2, and RB1CC1. Network analysis of these three core genes identified three related TFs (ATF4, DDIT3, and E2F1) and 31 miRNAs (**Supplementary Figure S4A**). Network analysis of core genes with RBPs was also conducted (**Supplementary Figure S4B**).

### 3.10 Immunoassay of core genes

**Figure 11:**
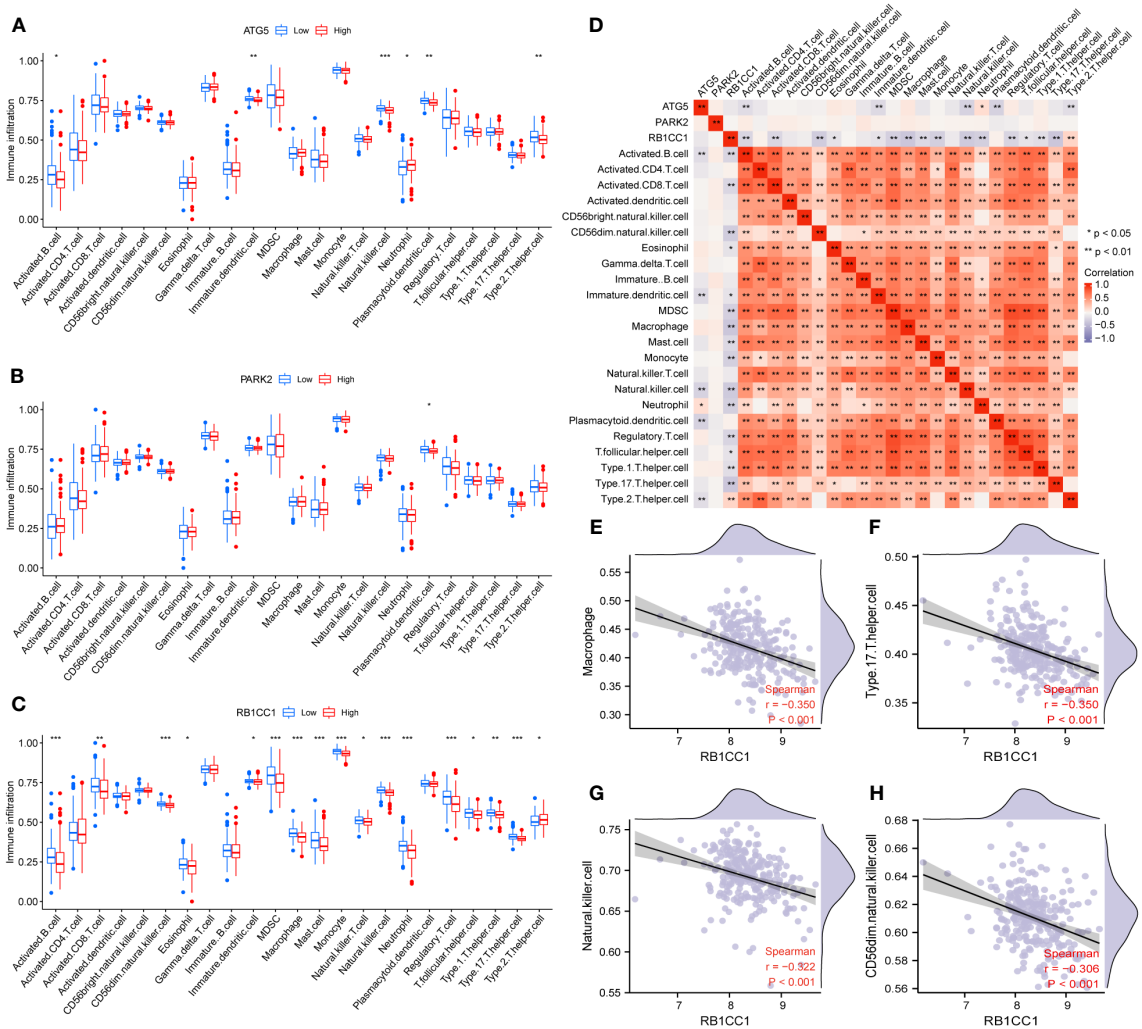
Immunoassay of core genes. **(A–C)** Differences in the infiltration of immune cells between different expression levels of the core genes including ATG5 **(A)**, PARK2 **(B)** and RB1CC1 **(C)**. **(D)** Correlation heatmap among core genes and 23 immune cells. Red represents a positive correlation and purple represents a negative correlation. **(E–H)** Matches with high correlation coefficients were selected for display. * P < 0.05, ** P < 0.01, and *** P < 0.001.

To investigate whether the core genes influenced the progression of liver fibrosis through immunity, we determined the association between immune cell infiltration levels and the expression of the core genes. ATG5 expression was associated with activated B cell, immature dendritic cell, natural killer cell, neutrophil, plasmacytoid dendritic cell, and type 2 T helper cell infiltration; PARK2 expression was associated with plasmacytoid dendritic cell infiltration; and RB1CC1 expression was associated with activated B cell, activated CD8 T cell, CD56dim natural killer cell, eosinophil, immature dendritic cell, myeloid-derived suppressor cell, macrophage, mast cell, monocyte, natural killer cell, natural killer cell, neutrophil, regulatory T cell, T follicular helper (Tfh) cell, type 1 T helper cell, Th17 cell, and type 2 T helper cell infiltration (**Figures 11A–C**). We assessed the correlations among the three core genes and 23 immune cells (**Figure 11D**), and related core genes and immune cells were screened based on r > 0.300 and P < 0.001. The expression of RB1CC1 was negatively correlated with macrophage (r = 0.350, P < 0.001), Th17 cell (r = 0.350, P < 0.001), natural killer cell (r = 0.322, P < 0.001), and CD56dim natural killer cell infiltration (r = 0.306, P < 0.001) (**Figures 11E–H**).

### 3.11 Expression of core genes in the mouse liver fibrosis model

**Figure 12:**
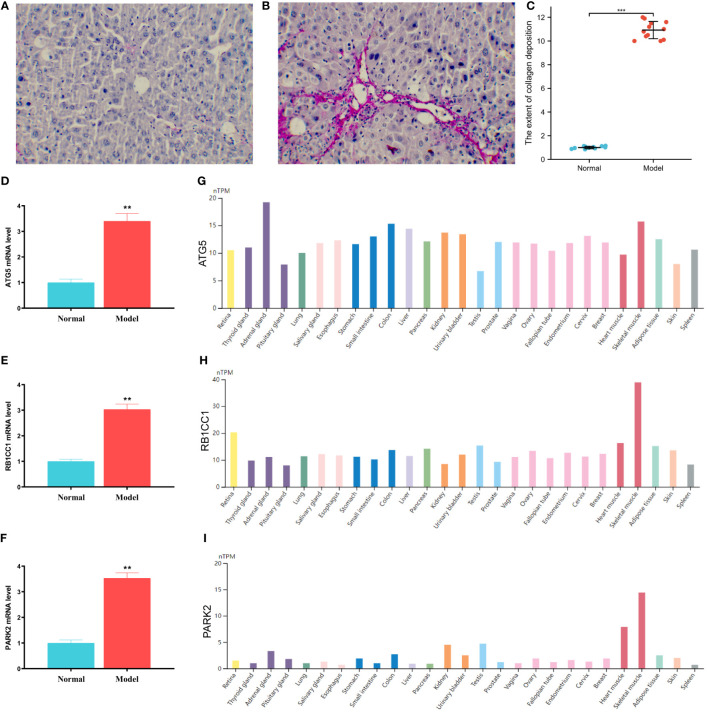
Expressions of the core genes in mice with liver fibrosis. **(A–C)** Sirius red staining of mouse liver tissue. A is normal group, B is model group, and C is semi-quantitative analysis of fiber bundles in mouse liver tissue. **(D–F)** The expression levels of ATG5, RB1CC1 and PARK2 in mouse liver tissue. **(D)** ATG5, **(E)** RB1CC1, **(F)** PARK2. **(G–I)** The expression levels of ATG5, RB1CC1 and PARK2 in different human tissues. **(G)** ATG5, **(H)** RB1CC1, **(I)** PARK2. ** P < 0.01, and *** P < 0.001.

In comparison to the normal group, the model group displayed a higher quantity of collagen fiber bundles, thereby validating the successful establishment of the HF model (**Figures 12A–C**). The mRNA levels of ATG5, RB1CC1, and PARK2 were found to be elevated in the model group relative to the normal group (**Figures 12D–F**). Utilizing the HPA database, the expression of core genes across different tissues was visualized. The findings indicated that ATG5 and RB1CC1 were highly expressed in human liver tissue. Although PARK2 was also present in human liver tissue, its expression was comparatively low (**Figures 12G–I**). These results underscored that ATG5, RB1CC1, and PARK2 were aberrantly expressed in the HF mouse model, aligning with our bioinformatics analysis. This suggests that these genes may play a crucial role in the pathogenesis of HF.

### 3.12 Immunoinfiltration levels of core genes in mouse liver fibrosis model

**Figure 13:**
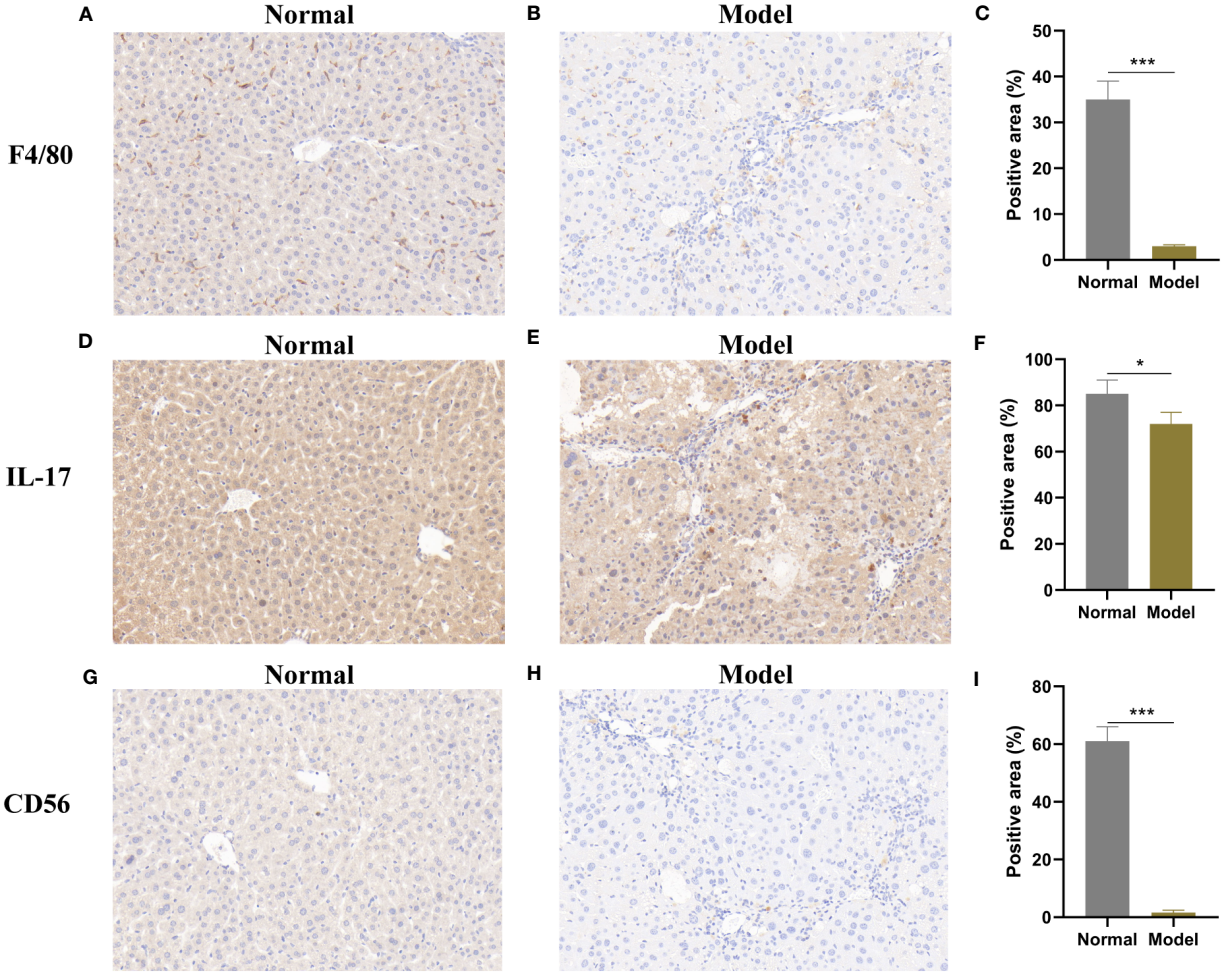
Immunoinfiltration levels of core genes in mouse liver fibrosis model. Immunohistochemical staining of F4/80 **(A–C)**, IL-17 **(D–F)** and CD56 **(G–I)**. Normal group: **(A, D, G)**; Model group: **(B, E, H); C, F, I** are semi-quantitative analysis of positive area. * P < 0.05, and *** P < 0.001.

To verify the association of RB1CC1 with immune cells, we examined the levels of four key markers of immune cells (macrophage, Th17 cell, natural killer cell and CD56dim natural killer cell). Compared with the normal group, the model group showed low positive area in F4/80 (P < 0.001) and IL-17 (P < 0.05), suggesting the decreased level of macrophage and Th17 cell respectively (**Figures 13A–F**). CD56 is a symbol of natural killer cell. The results showed that CD56 revealed less positive area in the model group than that in the normal group (P < 0.001), indicating the reduced level of natural killer cell and CD56dim natural killer cell (**Figures 13G–I**). These results suggest that macrophage, Th17 cell, natural killer cell and CD56dim natural killer cell are down-expressed in the progression of hepatic fibrosis; Combined with the negative correlation between RB1CC1 and the above four kinds of immune cells, RB1CC1 may promote the progression of liver fibrosis by regulating macrophage, Th17 cell, natural killer cell and CD56dim natural killer cell.

### 3.13 Function of RB1CC1 in a cell model of liver fibrosis

**Figure 14:**
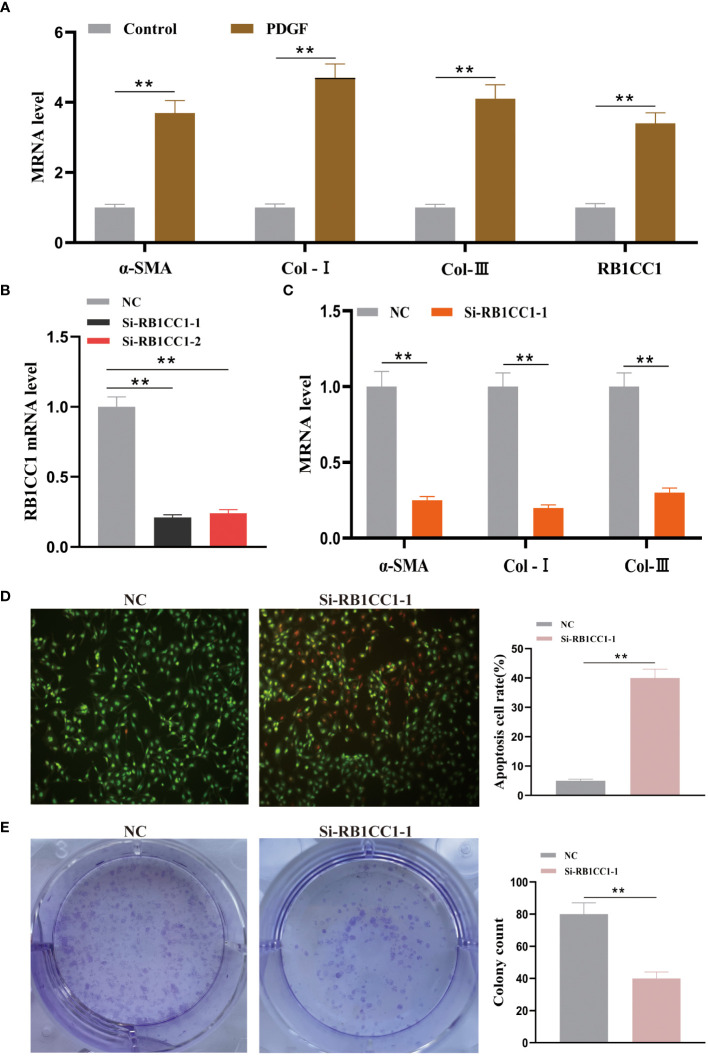
Effect of knocking down RB1CC1 on hepatic stellate cell activation. **(A)** The mRNA levels of α-SMA, Col-I, Col-III and RB1CC1 between the normal control and PDGF groups. **(B)** The RB1CC1 mRNA level of NC, si-RB1CC1-1 and si-RB1CC1-2 groups. **(C)** The mRNA levels of α-SMA, Col-I and Col-III between the NC and si-RB1CC1-1 groups. **(D)** The apoptosis cell rate between the NC and si-RB1CC1-1 groups. **(E)** The colony count between the NC and si-RB1CC1-1 groups. ** P < 0.01.

To further validate the bioinformatics results, we also conducted *in vitro* experiments. As shown in **Figure 14A**, compared with the normal group, the PDGF group revealed higher mRNA expressions in α-SMA, Col-I and Col-III (P < 0.01), indicating that cellular liver fibrosis model was successfully constructed. And the RB1CC1 mRNA expression in the PDGF group was higher than that in the normal group (P < 0.01), which is consistent with *in vivo* results. **Figure 14B** showed the good effect of silencing RBICC1 in LX2 cells. Si-RB1CC1 exhibited higher α-SMA, Col-I and Col-III mRNA levels and colony count, and lower apoptotic cell rate (P < 0.01), demonstrating that silenced RB1CC1 inhibited hepatic stellate cells activation (**Figures 14C–E**). Therefore, RB1CC1 may be a marker that promotes liver fibrosis.

### 3.14 RB1CC1 expression in patients with liver fibrosis

**Figure 15:**
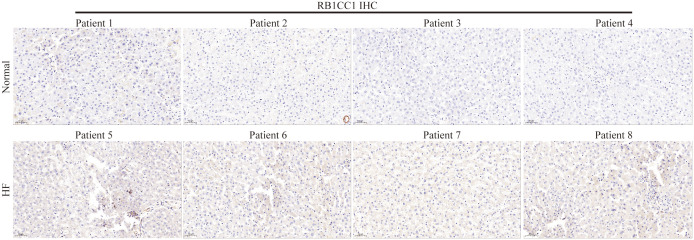
RB1CC1 expression in patients with liver fibrosis. Immunohistochemical staining of RB1CC1 in the HF patients and normal group.

The tissue microarray analysis suggested that RB1CC1 protein levels were also upregulated in liver fibrosis tissues compared to normal tissues (**Figure 15**), a finding that aligns with previous animal and cell experiments. Thus, *in vitro*, *in vivo*, and tissue multi-level data support our main findings that RB1CC1 is abnormally high expressed in liver fibrosis and could potentially serve as a promising biomarker for this disease.

## 4 Discussion

HF is the intermediary phase of chronic liver disease before cirrhosis and liver cancer, and currently has no effective treatment. In this study, using a combined GEO dataset, we identified 69 ARDEGs that were closely related to immunity and autophagy—important processes in HF progression. We used comprehensive bioinformatics methods to investigate the role of immune cell infiltration in HF. The HF risk model was constructed using the RF method and showed good prediction ability. Three core genes, ATG5, PARK2, and RB1CC1, were identified by PPI network and risk model analyses. Moreover, we confirmed the involvement of ATG5, PARK2, and RB1CC1 in HF in a mouse liver fibrosis model, among which RB1CC1 was never before reported in liver fibrosis. More importantly, knockdown of RB1CC1 in a cell model has been shown to inhibit hepatic stellate cell activation and alleviate liver fibrosis. This study further reveals the mechanism of autophagy and immunity in HF and provides new targets for the treatment of HF.

Bioinformatic immune analysis showed that there were significant differences in immune cell infiltration between the HC and HF groups. Specifically, the HF group showed a high expression of activated CD4 T cells, CD56bright natural killer cells, and CD56dim natural killer cells, but a low expression of eosinophils, macrophages, mast cells, neutrophils, and Th17 cells. Macrophages play an essential role in the pathogenesis of chronic liver injury. There are two main types of liver macrophages: resident macrophages in the hepatic sinuses called Kupffer cells and monocyte-derived macrophages, among which bone marrow-derived macrophages are the best described (40). Macrophage activity is integral to human innate immunity. The membrane receptors of the macrophages mediate important immunomodulatory responses, and promote or inhibit inflammatory effects (41). The different effects of macrophages are related to their phenotype (2). During liver injury, when macrophages present the Ly6Clo phenotype, the expression of matrix metalloproteinase inhibitor-1 is decreased and that of matrix metalloproteinase 2 is increased, which contributes to the dissolution of collagen and alleviates fibrosis (42). Kupfer cells promote mast cell proliferation by producing IL-18, so mast cells are subsequently reduced during fibrosis (43). CD4 T cells are also involved in liver fibrogenesis and have many subtypes, such as Th1, Th2, Th17, regulatory T (Treg), and Tfh cells. The total CD4 T cell count was elevated in fibrotic tissues, but the numbers of some subtypes showed the opposite trend (44). One of the characteristics of HF progression is the disorder of Th17 cells, which also represents an effective drug target for liver fibrosis (45). Neutrophils have anti-inflammatory properties and protective effects in both bile duct ligation and CCL4-induced liver fibrosis (46). And eosinophil-mediated liver protection was observed *in vivo* study (47). The increase of natural killer cells indicates that there are more necrotic cells that need to be removed during HF (48). These changes in immune cells eventually lead to a disorder of immune system homeostasis. Therefore, our results are consistent with those in previous studies and emphasize the importance of these immune cells in the pathogenesis of HF.

In determining the genes with the greatest influence on HF, we identified eight genes using the HF risk model (based on the RF method) and 20 genes by PPI network analysis. The core genes ATG5, RB1CC1, and PARK2 were verified *in vivo* analysis: The expression levels of ATG5, RB1CC1, and PARK2 in CCL4-induced HF mouse tissues were significantly higher than those in the control group, implicating these genes in the development of HF and validating the results of the bioinformatics analysis.

ATG5 is a common autophagy gene and its extensive participation in HF is widely reported. In the present study, ATG5 expression was downregulated in the HF group, while the HF mouse tissue group showed elevated ATG5 levels. This is likely due to the dual role of autophagy in the pathogenesis of HF. It has been reported that CCL4- or TAA-induced liver injury increases ATG5 expression and thereby enhances autophagy levels; meanwhile, loss of autophagic function through ATG5 knockdown in hepatic stellate cells and mice reduced fibrogenesis, consistent with the results of our experimental analysis (49–51). In contrast, some herbal monomers have been shown to alleviate liver fibrosis by upregulating ATG5, and overexpression of ATG5 could promote drug-induced inhibition of hepatic stellate cell activation and liver fibrosis, which corresponds to the results of our bioinformatics analysis (52, 53). Autophagy has emerged as a complex regulator whose profibrotic and antifibrotic properties depend on liver cell type, disease stage, and alterations in the immune microenvironment (54). Our study emphasizes the important role of autophagy in fibrosis and illustrates the bidirectional regulation of ATG5 in HF.

Growing evidence supports the involvement of PARK2 in HF progression, which is consistent with the results of our bioinformatic analysis and experimental validation revealing that PARK2 was upregulated in the HF group. PARK2, also known as parkin, is an E3 ubiquitin protein ligase and marker of mitophagy activation (55). PM2.5 can upregulate PARK2 levels by increasing reactive oxygen species production and promoting liver fibrosis (56, 57). Wu et al. showed that the T-cell immunoglobulin domain and mucin domain-4 could inhibit the activation of PARK2 and reduce the secretion of TGF-β1 to alleviate liver fibrosis (58). These and our findings suggest a role for PARK2 in promoting HF. However, other studies have suggested that PARK2-related mitophagy has anti-fibrotic effects (59, 60). This variation is likely related to differences in animal and hepatocyte models.

To the best of our knowledge, this is the first study to report the expression of RBICC1 in HF and the relationship between RB1CC1 and immune cell infiltration. In this study, the HF group exhibited higher levels of RB1CC1 than the control group did. The roles of RB1CC1 in liver diseases have only been studied in hepatocellular carcinoma (HCC). Specifically, RB1CC1 has been associated with migration and invasion ability in HCC, and RB1CC1-related signaling pathways sensitized tumor cells to ferroptosis, demonstrating that targeting RB1CC1 could promote the treatment of HCC (61, 62). RB1CC1 is abnormally expressed in both liver fibrosis and liver cancer, indicating that RB1CC1 is also a marker of liver fibrosis development into liver cancer. In the current study, RB1CC1 expression was negatively correlated with macrophage, Th17 cell, natural killer cell, and CD56dim natural killer cell infiltration. We found that the expression of four kinds of immune cell markers was reduced in the animal model of liver fibrosis, which verified the abnormal changes of macrophage, Th17 cell, natural killer cell, and CD56dim natural killer cell in the process of hepatic fibrosis. RB1CC1 may induce immunosuppression in the liver by affecting the infiltration of these immune cells. More interestingly, when RB1CC1 was knocked down in LX2 cells, collagen deposition and proliferation of LX2 cells were decreased, apoptosis was increased, and hepatic stellate cell activation was reduced. It is suggested that RB1CC1 could relieve liver fibrosis. Therefore, both *in vitro* and *in vivo* experiments confirmed RB1CC1 as a candidate molecular target for HF, and may participate in the occurrence and development of HF by regulating immune cell infiltration.

Although our study elucidates the molecular biomarkers and potential mechanisms of HF progression, it has some limitations. On the one hand, future studies should investigate multicenter population-wide datasets. On the other hand, because the data were obtained from public databases, our analysis could have been affected by an inherent bias.

In conclusion, the present study elucidates the autophagy characteristics and immune cell profiles of HF and identifies biomarkers of liver fibrosis. This is the first study to identify RB1CC1 in HF, which may influence immune cell infiltration and aggravate liver fibrosis. Our findings shed new light on molecular pathogenesis and diagnosis biomarkers of HF.

## Data availability statement

The datasets presented in this study can be found in online repositories. The names of the repository/repositories and accession number(s) can be found in the article/**Supplementary Material**.

## Ethics statement

The animal study was approved by The Animal Care & Welfare Committee of Guangxi Medical University. The study was conducted in accordance with the local legislation and institutional requirements.

## Author contributions

HW: Conceptualization, Project administration, Resources, Supervision, Writing – review & editing. YH: Conceptualization, Formal Analysis, Software, Validation, Writing – original draft, Writing – review & editing. WL: Data curation, Formal Analysis, Software, Writing – original draft, Writing – review & editing. ZY: Data curation, Validation, Writing – review & editing. TL: Validation, Writing – original draft. XW: Project administration, Resources, Writing – review & editing.
