# Supplementary material for: Machine learning and experimental validation identified autophagy signature in hepatic fibrosis
Source: Front Immunol. 2024 Feb 28;15:1337105. doi: 10.3389/fimmu.2024.1337105 (PMC10933073; doi:10.3389/fimmu.2024.1337105)
Supplement: Supplementary file 1 [file DataSheet_1.docx]

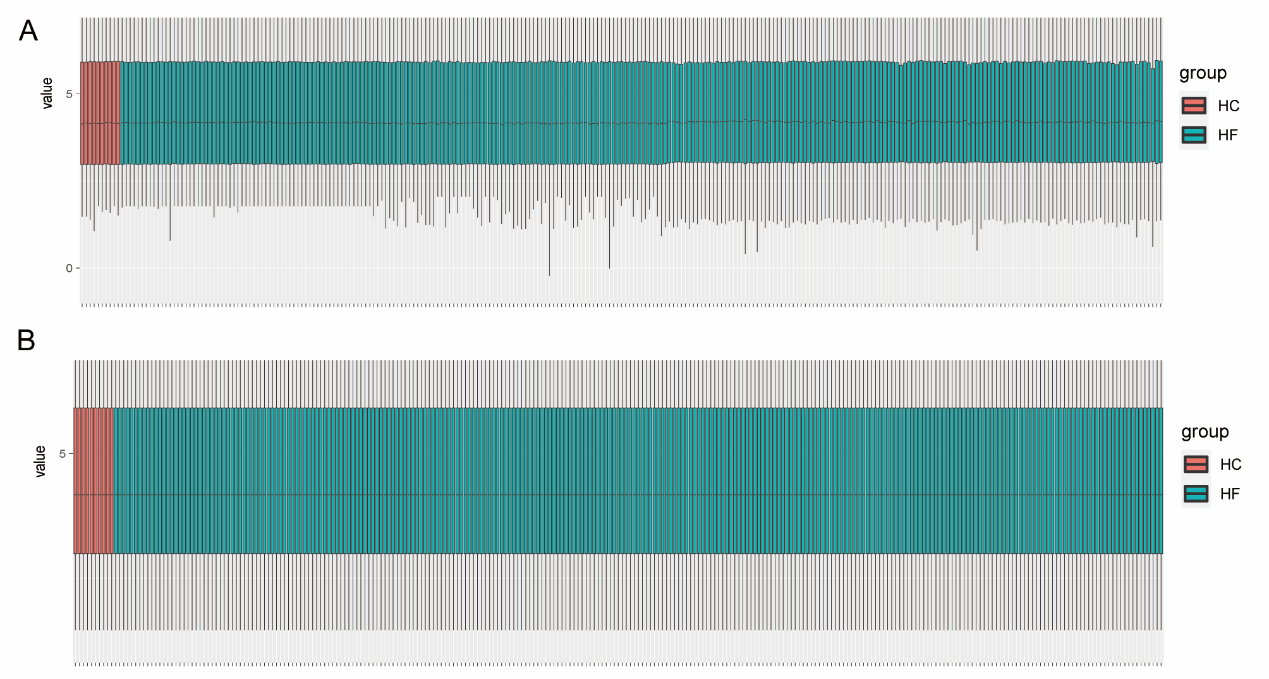


**Figure S1 Standardization of datasets.** A. Distribution of expression profiles of three datasets before standardization. B. Distribution of expression profiles of three datasets after standardization.


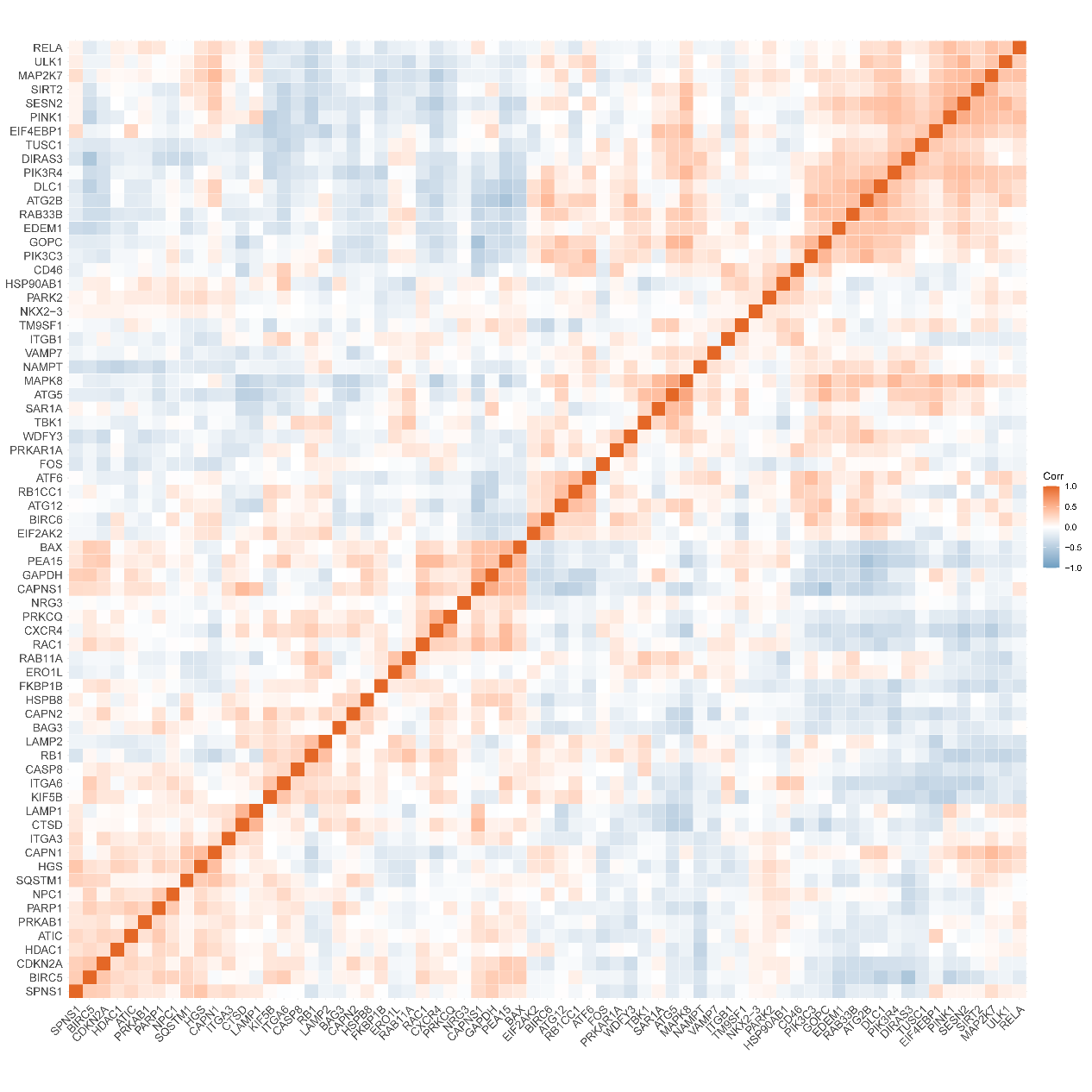


**Figure S2 Correlation heatmap among ARDEGs.** The higher the correlation, the darker the color.


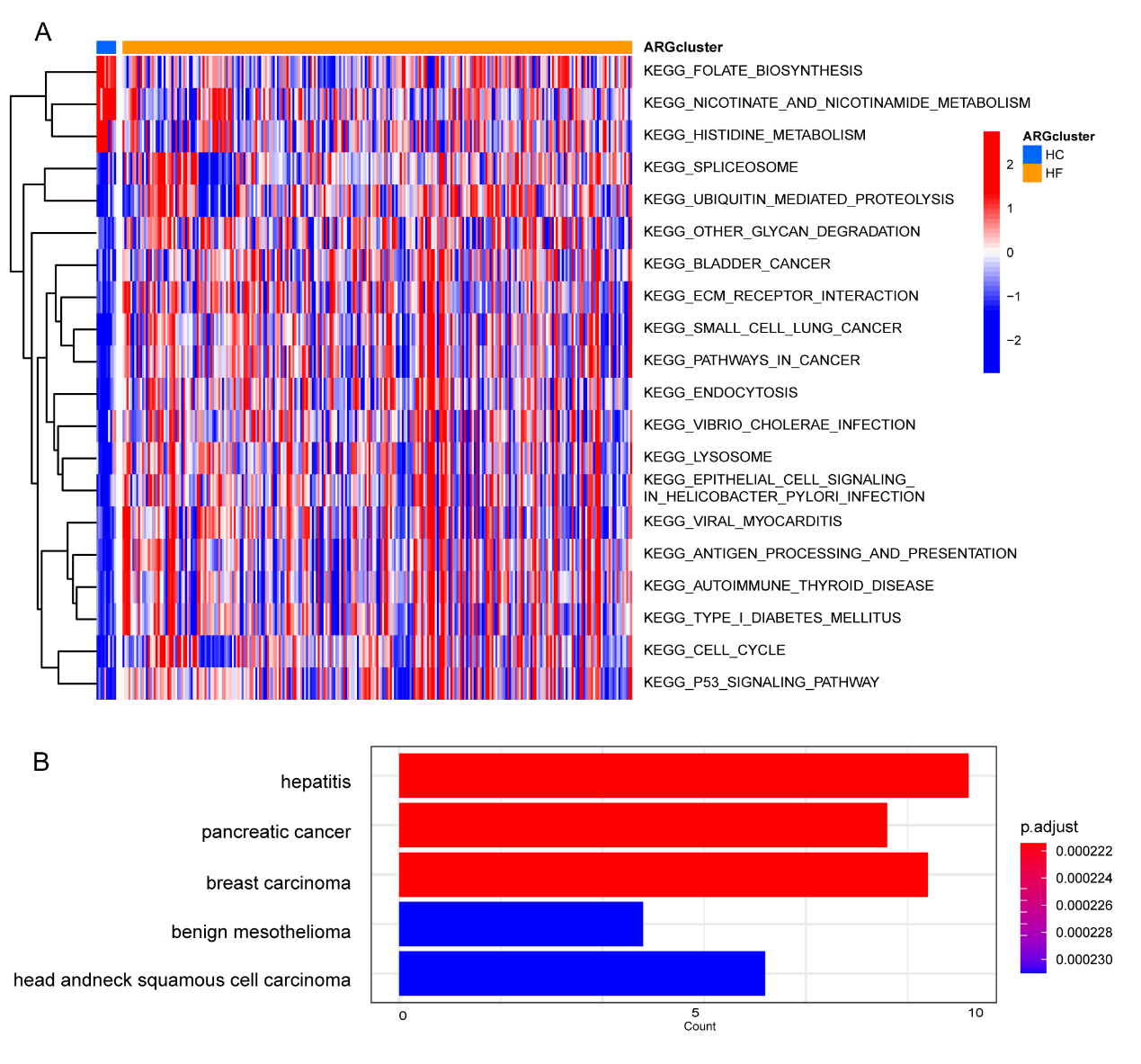


**Figure S3 GSVA and DO analysis.** A. Heatmap of major biological functions and pathways based on GSVA analysis of integrated datasets. B. ARDEGs primarily enriched in disease pathways including hepatitis, pancreatic cancer, breast carcinoma, benign mesothelioma, head and neck squamous cell carcinoma.


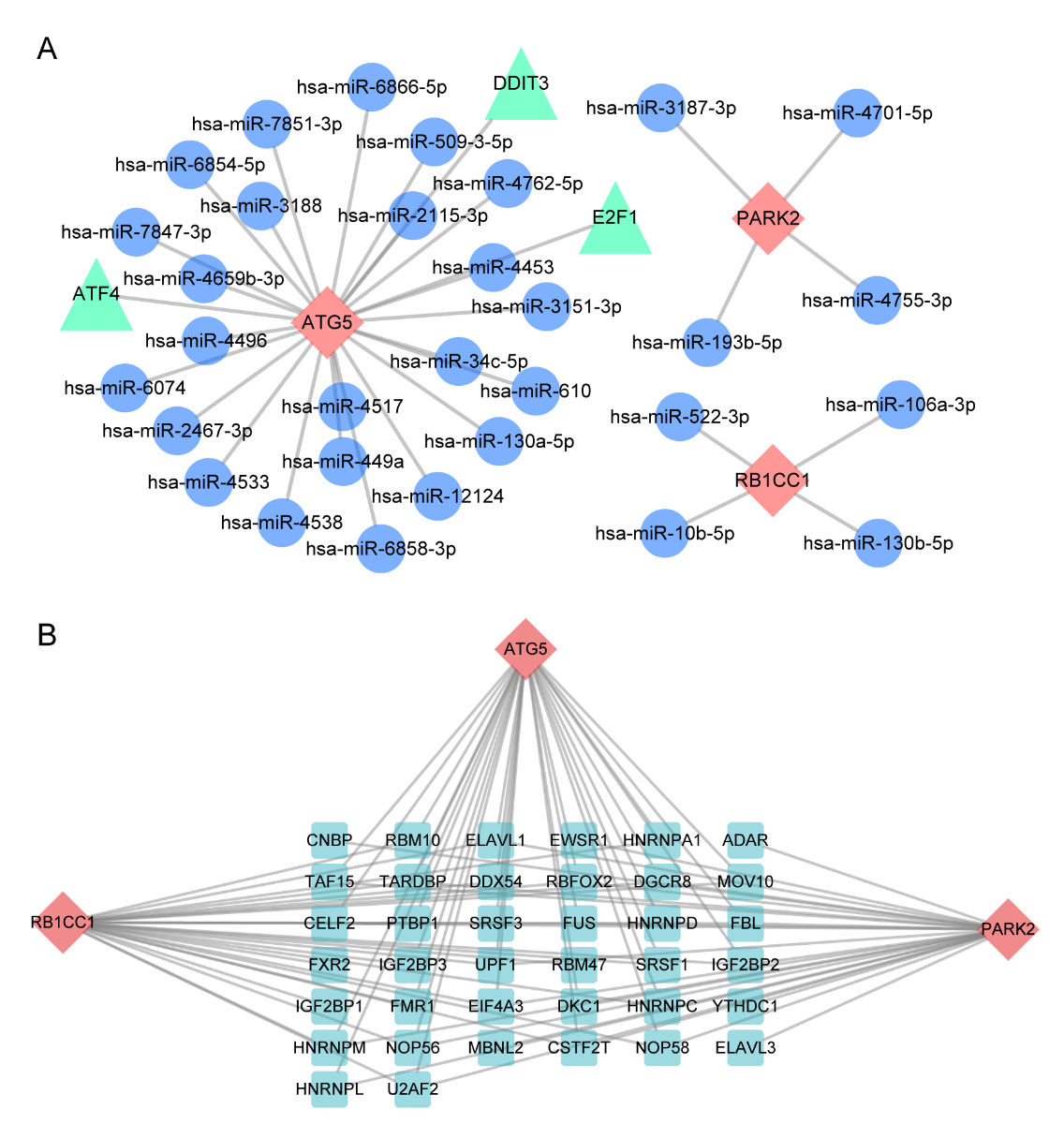


**Figure S4 Network analysis of core genes with miRNA, TF, and RBP.** A. Network analysis of core genes with miRNA and TF. Red nodes represent core genes, green nodes indicate TF, and Dark blue nodes show miRNA. B. Network analysis of core genes with RBP, Red nodes represent core genes, and light blue shows RBP.
