# Supplementary material for: Machine learning and experimental validation identified autophagy signature in hepatic fibrosis
Source: Front Immunol. 2024 Feb 28;15:1337105. doi: 10.3389/fimmu.2024.1337105 (PMC10933073; doi:10.3389/fimmu.2024.1337105)
Supplement: Supplementary file 2 [file DataSheet_2.docx]

Table S1 The information of GEO datasets

| ID | GEO number | Platforms | Sample | Source types | Reference |
| --- | --- | --- | --- | --- | --- |
| 1 | GSE6764 | GPL570 | 10HC, 65HF | liver tissue | Wurmbach et.al [21] |
| 2 | GSE49541 | GPL570 | 72HF | liver tissue | Moylan et.al  [22; 23] |
| 3 | GSE84044 | GPL570 | 124HF | liver tissue | Wang et.al  [24] |

Table S2 The sequences of primers used for RT-PCR

| Genes | Forward primer (5′-3′) | Reverse primer (5′-3′) |
| --- | --- | --- |
| ATG5 | TGCATCAAGTTCAGCTCTTCC | ACTGGTCAAATCTGTCATTCTGC |
| PARK2 | GAGGTCGATTCTGACACCAGC | CCGGCAAAAATCACACGCAG |
| RB1CC1 | GACACTGAGCTAACTGTGCAA | GCGCTGTAAGTACACACTCTTC |
| Beta-actin  Col-I  Col-III  α-SMA  RB1CC1 | CAGCCTTCCTTCTTGGGTAT  GTTGCTGCTTGCAGTAACCTT  GGAGCTGGCTACTTCTCGC  AAAAGACAGCTACGTGGGTGA  ATCGAAGAGTGTGTACCTACAGT | TGGCATAGAGGTCTTTACGG  AGGGCCAAGTCCAACTCCTT  GGGAACATCCTCCTTCAACAG  GCCATGTTCTATCGGGTACTTC  GCAGGTGGACGATCACATAAGAT |

Table S3 GO analysis of ARDEGs

| ONTOLOGY | ID | Description | GeneRatio | BgRatio | pvalue | p.adjust | qvalue |
| --- | --- | --- | --- | --- | --- | --- | --- |
| BP | GO:0016236 | macroautophagy | 23/66 | 295/18670 | 2.97e-25 | 7.52e-22 | 4.82e-22 |
| BP | GO:0006914 | autophagy | 26/66 | 496/18670 | 3.49e-24 | 2.94e-21 | 1.89e-21 |
| BP | GO:0061919 | process utilizing autophagic mechanism | 26/66 | 496/18670 | 3.49e-24 | 2.94e-21 | 1.89e-21 |
| BP | GO:0016241 | regulation of macroautophagy | 13/66 | 171/18670 | 2.70e-14 | 1.71e-11 | 1.10e-11 |
| BP | GO:0061912 | selective autophagy | 9/66 | 49/18670 | 8.99e-14 | 4.55e-11 | 2.92e-11 |
| CC | GO:0005776 | autophagosome | 10/66 | 93/19717 | 5.63e-13 | 1.57e-10 | 8.48e-11 |
| CC | GO:0000407 | phagophore assembly site | 7/66 | 32/19717 | 1.07e-11 | 1.49e-09 | 8.05e-10 |
| CC | GO:0016234 | inclusion body | 8/66 | 82/19717 | 2.98e-10 | 2.77e-08 | 1.50e-08 |
| CC | GO:0045335 | phagocytic vesicle | 9/66 | 132/19717 | 5.50e-10 | 3.84e-08 | 2.07e-08 |
| CC | GO:0034045 | phagophore assembly site membrane | 5/66 | 16/19717 | 1.53e-09 | 8.53e-08 | 4.60e-08 |
| MF | GO:0044389 | ubiquitin-like protein ligase binding | 10/64 | 308/17697 | 1.45e-07 | 1.94e-05 | 1.29e-05 |
| MF | GO:0042826 | histone deacetylase binding | 7/64 | 111/17697 | 1.46e-07 | 1.94e-05 | 1.29e-05 |
| MF | GO:0031625 | ubiquitin protein ligase binding | 9/64 | 290/17697 | 9.42e-07 | 8.35e-05 | 5.55e-05 |
| MF | GO:0004674 | protein serine/threonine kinase activity | 10/64 | 439/17697 | 3.64e-06 | 2.42e-04 | 1.61e-04 |
| MF | GO:0001618 | virus receptor activity | 5/64 | 74/17697 | 7.01e-06 | 3.11e-04 | 2.07e-04 |

Table S4 KEGG enrichment analysis of ARDEGs

| ONTOLOGY | ID | Description | GeneRatio | BgRatio | pvalue | p.adjust | qvalue |
| --- | --- | --- | --- | --- | --- | --- | --- |
| KEGG | hsa04140 | Autophagy - animal | 15/55 | 137/8076 | 8.52e-15 | 1.49e-12 | 7.44e-13 |
| KEGG | hsa05131 | Shigellosis | 15/55 | 246/8076 | 4.73e-11 | 4.14e-09 | 2.07e-09 |
| KEGG | hsa05022 | Pathways of neurodegeneration - multiple diseases | 18/55 | 475/8076 | 9.76e-10 | 5.69e-08 | 2.84e-08 |
| KEGG | hsa05010 | Alzheimer disease | 15/55 | 369/8076 | 1.33e-08 | 5.82e-07 | 2.91e-07 |
| KEGG | hsa04210 | Apoptosis | 10/55 | 136/8076 | 2.02e-08 | 7.07e-07 | 3.53e-07 |

Table S5 GSEA of ARDEGs

| Description | enrichmentScore | NES | pvalue | p.adjust | qvalues |
| --- | --- | --- | --- | --- | --- |
| REACTOME_CELL_CYCLE_MITOTIC | -0.54444 | -2.26763 | 0.001792 | 0.032883 | 0.025272 |
| REACTOME_SIGNALING_BY_RHO_GTPASES | -0.41912 | -1.70746 | 0.001835 | 0.032883 | 0.025272 |
| REACTOME_M_PHASE | -0.52794 | -2.13321 | 0.001838 | 0.032883 | 0.025272 |
| REACTOME_TRANSCRIPTIONAL_REGULATION_BY_TP53 | -0.41764 | -1.69221 | 0.001838 | 0.032883 | 0.025272 |
| KEGG_FOCAL_ADHESION | -0.43357 | -1.64931 | 0.001855 | 0.032883 | 0.025272 |
| REACTOME_MRNA_SPLICING | -0.42724 | -1.6122 | 0.001855 | 0.032883 | 0.025272 |
| REACTOME_INTERFERON_ALPHA_BETA_SIGNALING | -0.76847 | -2.50344 | 0.001862 | 0.032883 | 0.025272 |
| WP_MIRNA_REGULATION_OF_DNA_DAMAGE_RESPONSE | -0.61107 | -1.99067 | 0.001862 | 0.032883 | 0.025272 |
| PID_AVB3_INTEGRIN_PATHWAY | -0.60705 | -1.99885 | 0.001869 | 0.032883 | 0.025272 |
| REACTOME_MITOTIC_PROMETAPHASE | -0.56199 | -2.13539 | 0.001873 | 0.032883 | 0.025272 |
| REACTOME_SIGNALING_BY_ROBO_RECEPTORS | -0.43492 | -1.65255 | 0.001873 | 0.032883 | 0.025272 |
| REACTOME_INTERFERON_SIGNALING | -0.63141 | -2.39144 | 0.00188 | 0.032883 | 0.025272 |
| REACTOME_MITOTIC_G2_G2_M_PHASES | -0.49711 | -1.88278 | 0.00188 | 0.032883 | 0.025272 |
| REACTOME_SEPARATION_OF_SISTER_CHROMATIDS | -0.60558 | -2.28117 | 0.001883 | 0.032883 | 0.025272 |
| REACTOME_COLLAGEN_DEGRADATION | -0.64545 | -2.08891 | 0.001887 | 0.032883 | 0.025272 |
| WP_DNA_DAMAGE_RESPONSE | -0.61227 | -1.98155 | 0.001887 | 0.032883 | 0.025272 |
| KEGG_ANTIGEN_PROCESSING_AND_PRESENTATION | -0.6898 | -2.28461 | 0.00189 | 0.032883 | 0.025272 |
| KEGG_VIRAL_MYOCARDITIS | -0.59624 | -1.93167 | 0.00189 | 0.032883 | 0.025272 |
| PID_INTEGRIN1_PATHWAY | -0.67889 | -2.19943 | 0.00189 | 0.032883 | 0.025272 |
| REACTOME_EXTRACELLULAR_MATRIX_ORGANIZATION | -0.42763 | -1.69793 | 0.00189 | 0.032883 | 0.025272 |
| REACTOME_HIV_INFECTION | -0.42881 | -1.63833 | 0.00189 | 0.032883 | 0.025272 |
| REACTOME_INFLUENZA_INFECTION | -0.49947 | -1.82907 | 0.00189 | 0.032883 | 0.025272 |
| REACTOME_APC_C_MEDIATED_DEGRADATION_OF_CELL_CYCLE_PROTEINS | -0.61869 | -2.0987 | 0.001894 | 0.032883 | 0.025272 |
| REACTOME_CELL_CYCLE_CHECKPOINTS | -0.61216 | -2.36849 | 0.001894 | 0.032883 | 0.025272 |
| REACTOME_INTEGRIN_CELL_SURFACE_INTERACTIONS | -0.59492 | -2.01807 | 0.001894 | 0.032883 | 0.025272 |
| REACTOME_MITOTIC_G1_PHASE_AND_G1_S_TRANSITION | -0.59766 | -2.20413 | 0.001894 | 0.032883 | 0.025272 |
| REACTOME_MITOTIC_METAPHASE_AND_ANAPHASE | -0.59663 | -2.28269 | 0.001894 | 0.032883 | 0.025272 |
| REACTOME_PROCESSING_OF_CAPPED_INTRON_CONTAINING_PRE_MRNA | -0.44547 | -1.70866 | 0.001894 | 0.032883 | 0.025272 |
| REACTOME_REGULATION_OF_EXPRESSION_OF_SLITS_AND_ROBOS | -0.45196 | -1.66679 | 0.001894 | 0.032883 | 0.025272 |
| WP_FOCAL_ADHESIONPI3KAKTMTORSIGNALING_PATHWAY | -0.34019 | -1.35904 | 0.011257 | 0.080112 | 0.061569 |
| WP_HIPPOYAP_SIGNALING_PATHWAY | -0.62056 | -1.64991 | 0.013861 | 0.086879 | 0.06677 |
| REACTOME_SIGNALING_BY_WNT | -0.34642 | -1.36336 | 0.015066 | 0.091426 | 0.070265 |
| REACTOME_AUTOPHAGY | -0.39802 | -1.45887 | 0.01518 | 0.091709 | 0.070482 |

Table S6 GSVA of ARDEGs

| id | logFC | AveExpr | t | P.Value | adj.P.Val |
| --- | --- | --- | --- | --- | --- |
| KEGG_NICOTINATE_AND_NICOTINAMIDE_METABOLISM | -0.343839508 | 0.009133469 | -5.139291494 | 5.22E-07 | 9.61E-05 |
| KEGG_ENDOCYTOSIS | 0.208186241 | -0.002025034 | 4.831578599 | 2.25E-06 | 0.000206829 |
| KEGG_FOLATE_BIOSYNTHESIS | -0.407012204 | -0.000857814 | -4.429878039 | 1.36E-05 | 0.000834115 |
| KEGG_SMALL_CELL_LUNG_CANCER | 0.28846435 | -0.010511879 | 4.306689504 | 2.30E-05 | 0.00106004 |
| KEGG_CELL_CYCLE | 0.308328856 | -0.0222009 | 3.862909915 | 0.000139634 | 0.004171271 |
| KEGG_LYSOSOME | 0.288744424 | -0.001020957 | 3.853521752 | 0.000144815 | 0.004171271 |
| KEGG_BLADDER_CANCER | 0.238257309 | -0.009093589 | 3.820867435 | 0.000164287 | 0.004171271 |
| KEGG_SPLICEOSOME | 0.271410936 | -0.019620686 | 3.795121591 | 0.00018136 | 0.004171271 |
| KEGG_ANTIGEN_PROCESSING_AND_PRESENTATION | 0.330095125 | -0.009347115 | 3.695360791 | 0.000264686 | 0.005411351 |
| KEGG_VIRAL_MYOCARDITIS | 0.346529613 | 0.00304293 | 3.645386765 | 0.00031891 | 0.005867951 |
| KEGG_PATHWAYS_IN_CANCER | 0.175063512 | -0.00485728 | 3.617182678 | 0.000353965 | 0.005920874 |
| KEGG_EPITHELIAL_CELL_SIGNALING_IN_HELICOBACTER_PYLORI_INFECTION | 0.229789302 | -0.00613272 | 3.560941231 | 0.000434951 | 0.006338352 |
| KEGG_UBIQUITIN_MEDIATED_PROTEOLYSIS | 0.203348617 | -0.008261349 | 3.55292563 | 0.000447818 | 0.006338352 |
| KEGG_ECM_RECEPTOR_INTERACTION | 0.28700378 | 0.002462363 | 3.478646415 | 0.000585269 | 0.007692101 |
| KEGG_AUTOIMMUNE_THYROID_DISEASE | 0.333806214 | -0.014135704 | 3.390549948 | 0.000799273 | 0.009398301 |
| KEGG_P53_SIGNALING_PATHWAY | 0.241938392 | -0.004386504 | 3.384194924 | 0.000817244 | 0.009398301 |
| KEGG_TYPE_I_DIABETES_MELLITUS | 0.370909822 | -0.003196773 | 3.358436439 | 0.000894012 | 0.00967636 |
| KEGG_HISTIDINE_METABOLISM | -0.275124086 | 0.002938134 | -3.328427711 | 0.000991904 | 0.010139468 |
| KEGG_VIBRIO_CHOLERAE_INFECTION | 0.209151218 | -0.007576839 | 3.272715798 | 0.001200591 | 0.011626772 |
| KEGG_OTHER_GLYCAN_DEGRADATION | 0.270856292 | 0.00036957 | 3.186187143 | 0.001606882 | 0.014783313 |
